# Study of Resonance between Bogie Hunting and Carbody Mode via Field Measurements and Dynamic Simulation

**DOI:** 10.3390/s24165194

**Published:** 2024-08-11

**Authors:** Sheng Yang, Fansong Li, Pingbo Wu, Jijun Gong

**Affiliations:** 1 State Key Laboratory of Rail Transit Vehicle System, Southwest Jiaotong University, Chengdu 610031, China; shyang@jsut.edu.cn (S.Y.); wupingbo@163.com (P.W.); 18384124476@163.com (J.G.); 2 School of Automotive and Traffic Engineering, Jiangsu University of Technology, Changzhou 213001, China; 3 China Railway Materials Operation and Maintenance Technology Co., Ltd., Beijing 100070, China

**Keywords:** field test, diamond mode, bogie hunting, flexible carbody, resonance analysis

## Abstract

By addressing the phenomenon of carbody abnormal vibrations in the field, the acceleration of the carbody and bogie was measured using accelerometers, and the diamond mode of the carbody was identified. The equivalent conicity of the wheelset and the acceleration at the frame end indicated that the shaking of the carbody was caused by bogie hunting. In the SIMPACK simulation, the acceleration frequency and amplitude at the frame end and midsection of the side beam were calculated. The lateral deformation amplitude of the side beam in the finite element model was extracted, and a modal shape function for the diamond-shaped mode was established. By utilizing the modal vibration equation, the modal generalized forces of the carbody were computed, revealing that, during carbody shaking, the yaw damper force contributed significantly among the forces of the secondary suspension, with the phase difference between the front and rear bogies approaching 180°. This insight offers a novel perspective for subsequent active control strategies. Subsequently, these modal generalized forces were applied as external excitation to a coupled vibration model encompassing both the carbody and transformer. Aiming to reduce the acceleration amplitude at the side beam, the transformer was treated as a dynamic vibration absorber, allowing for the optimization of its lateral suspension parameters. As a result, the lateral and vertical acceleration amplitudes at the side beam were concurrently reduced, with the maximum decrease reaching 58.5%, significantly enhancing the ride comfort.

## 1. Introduction

The degradation in the ride comfort of electric multiple units (EMUs) stemming from the mode vibration of the carbody has garnered significant attention in recent years. The trend towards lightweight designs in carbody construction has led to a reduction in the frequency of the carbody’s low-order modes, while the escalating vehicle speed and the evolving wheel–rail contact have broadened the spectrum of excitation frequencies.

Carbody low-order modes specifically comprise the diamond mode (DM) and the vertical bending mode (VBM). Currently, the research focusing on mitigating the mode vibration of carbody has primarily centered around the VBM. The methods for suppressing VBM vibration include installing an anti-bending bar [1] and damper [2] on the underframe of the carbody, adopting a semi-active suspension [3,4] and active suspension [5,6], optimizing the longitudinal vibration transmission of the bogies and the carbody [7,8], and modifying the VBM nodes [9]. At the current stage, the design of suspension parameters for underframe equipment mainly focuses on the vertical direction, using a dynamic vibration absorption (DVA) design for suppressing the VBM vibration [10,11]. However, there is limited research on the suspension design of the equipment in other directions.

With the accumulation of EMU mileages and the deterioration of the wheel–rail relationship, the abnormal vibration behavior of carbody has attracted increasing attention. Abnormal vibrations occur on some sections of the track, with the dominant frequency of acceleration ranging from 8 to 10 Hz. The carbody experiences obvious vibration in both the lateral and vertical directions, and the abnormal vibration disappears after the vehicle decelerates. Distinguishing from the “carbody-swaying” behavior within 2 Hz caused by the resonance between carbody hunting and the rigid mode of the carbody [12,13], this abnormal vibration behavior is referred to as the “carbody-shaking” (CS) phenomenon. The duration of the vibration is 3–5 s, with noticeable shaking in places such as the luggage racks and VIP seats, causing significant discomfort to passengers. At the same time, the resonance shortens the fatigue life of the carbody structure [14].

Analyses of abnormal vibration issues during vehicle operation are often conducted by combining field measurements and a dynamic simulation [15,16,17]. Shi [18] analyzed the acceleration of a carbody during CS and found that the vibration exhibited characteristics of the DM. Meanwhile, the main frequency of the bogie and carbody vibration was consistent, and the bogie underwent a minor hunting motion. This indicated that the hunting of the bogie triggered the DM vibration. Wei [19] measured the modal characteristics of the carbody through a rig test, and reproduced CS in a dynamic simulation with measured wheel–rail profiles. The CS was eliminated by modifying the wheel profile and grinding the rail. Chang [20] studied the impact of turnout on CS. When passing through multiple turnouts, the vehicle was subject to continuous impacts, and the superposition effect of the vibration aggravated CS. After grinding the turnout profile, the problem was successfully solved.

The aforementioned research primarily explains the causes of CS, and the proposed solutions mainly include profiling wheels, grinding rails, and designing a new carbody structure to increase the DM frequency. These measures, however, come with an increase in the operational, maintenance, and design costs of the vehicle. From the perspective of enhancing the bogie-hunting stability, CS can be effectively suppressed through an increase in the stiffness of the primary longitudinal suspension and the yaw damper [21,22,23]. However, this method will compromise the stability of carbody hunting, and it poses a challenge for the vehicle’s suspension parameters to simultaneously fulfill the requirements of enhancing the stability of both carbody hunting and bogie hunting. The use of active control in the lateral direction of the bogie can improve its hunting stability [24,25], but it will increase the manufacturing costs and has not yet been applied to actual vehicles. Setting the motor as a DVA to keep the hunting frequency of the bogie away from the DM can significantly suppress the resonance [26,27], but it is not applicable to trailer cars and vehicles with body-suspended motors. Based on the acceleration transfer characteristics of the underframe equipment and the side beam during CS, Yang [28] decreased the amplitude of the carbody vibration by reducing the roll frequency of the equipment.

Currently, the research on CS is still in its initial stages, and is focusing primarily on describing the phenomenon and exploring the causes. However, the mechanism behind the coupled vibration of bogie hunting and DM vibration remains unclear. The current theoretical model for vehicle lateral vibration mainly focus on a bifurcation analysis of the vehicle system [29,30,31], and the DM is excluded. Thus, there is still a lack of a simple, effective, and widely applicable vibration reduction solution. While the design of DVA for underframe equipment in the vertical direction is both simple and effective, suspension design in other directions relies heavily on static and geometric principles. Furthermore, there are relatively few studies on the lateral coupled vibration between the carbody and the underframe equipment. Consequently, further research has primarily focused on semi-active and active suspension designs, but their feasibility and engineering application value remain questionable.

Through field measurements, this paper obtained the amplitude–frequency characteristics of the measuring points’ acceleration on the carbody and bogie, considering both normal operating conditions and CS conditions. By integrating a dynamic simulation and modal vibration equations, the contribution of secondary suspension forces to CS and the frequency–phase characteristics of various components were analyzed. Finally, the lateral suspension frequency of the underframe equipment was optimized. At present, the suspension parameters of the underframe equipment mainly meet its dynamic absorption of VBM vibration, and the lateral suspension stiffness depends on the physical characteristics of the rubber component. Optimizing the lateral suspension parameters of the equipment leads to lower manufacturing and operational costs and a broader applicability across vehicle types, without compromising the stability of carbody hunting.

The rest of this paper is organized as follows. Section 2 outlines the testing scheme for CS, and proceeds with a detailed analysis of the collected data. In Section 3, the phenomenon of CS is reproduced in SIMPACK, enabling a thorough examination of how the shaking frequency varies across various speeds. In Section 4, a theoretical model of modal lateral vibration, incorporating the DM, is formulated and the suppression effectiveness on CS in the SIMPACK simulation is primarily gauged by the reduction in the acceleration of the side beam.

## 2. Field Measurements for EMUs

As the mileage approached the design limit for the wheel re-profiling cycle, a specific type of high-speed EMU encountered CS while traveling at 350 km/h on a particular section of the Shanghai–Beijing line. Upon reversing its direction and passing through the same section, the vehicle experienced identical vibration. In response to this issue, a field test was conducted.

### 2.1. Methods for Field Test

**Figure 1:**
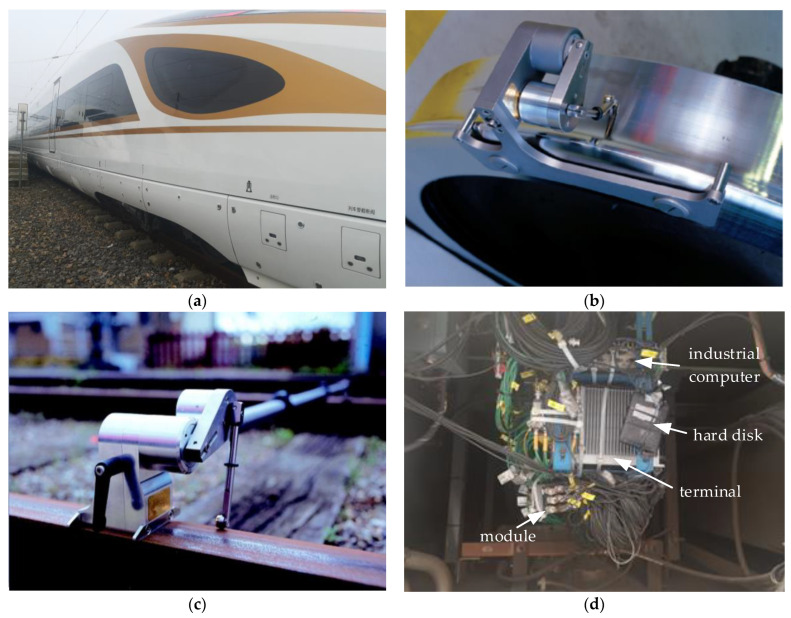
Overview of field test: (**a**) tested vehicle; (**b**) wheel profile measurement; (**c**) rail profile measurement; and (**d**) data-acquisition terminal.

**Figure 2:**
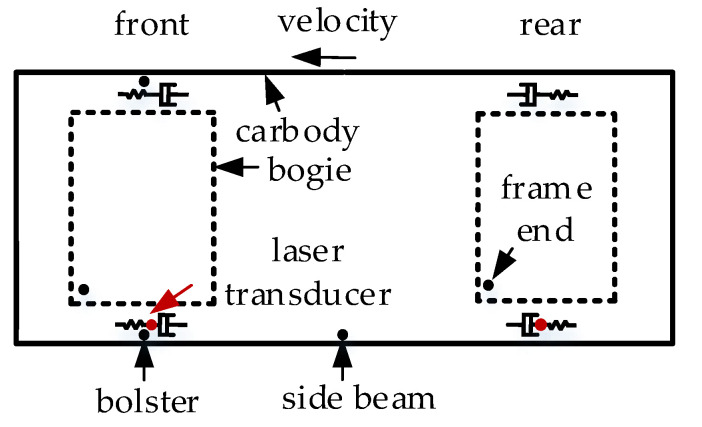
Location of measuring points.

**Figure 3:**
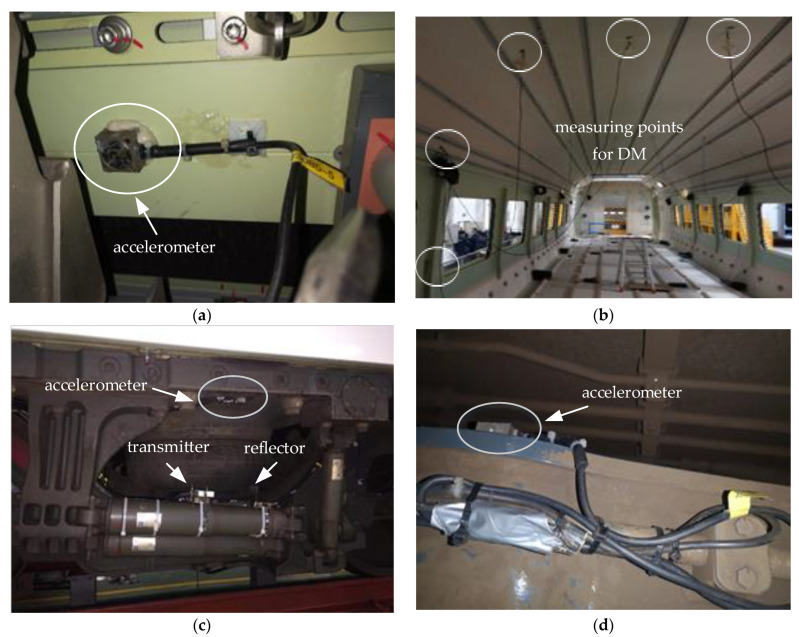
Installation of the sensors: (**a**) middle of carbody side beam; (**b**) passenger compartment; (**c**) bolster and YD; and (**d**) frame end.

The initial work of the field test was mainly divided into the measurement of the wheel–rail profile and the installation of sensors. The model of the rail-profile-measuring instrument employed in the test was MiniProf, featuring a sampling frequency of 0.1 mm. The rail profile in the section where the CS occurred was measured, as Figure 1c shows. There were two types of sensors used, namely triaxial accelerometers and laser transducers, and their specific parameters are listed in Table 1. Sensors for measuring acceleration were installed on the bolsters, side beams and frame ends, as shown in Figure 2. Sensors for measuring DM were installed on 7 sections inside the passenger compartment, as shown in Figure 3b. The laser transducer, comprising a transmitter mounted on the damper sleeve and a corresponding reflector at the opposite end, was utilized to obtain precise measurements of the displacement between these two components, as shown in Figure 3c. Sensors were connected to the data-acquisition terminal through different modules. Equipped with an industrial computer and a large-capacity hard disk, the terminal was firmly fixed on the vehicle equipment cabin.

Based on the vehicle’s forward direction, the bogies were differentiated into a front bogie and a rear bogie. For each bogie, two yaw dampers (YDs) were installed in parallel on the same side, with a laser transducer mounted on the upper one. Additionally, the YDs of the front and rear bogies were installed in opposite directions. The measuring range of the laser transducer was 30–130 mm, and the installation distance between the transmitter and reflector was 80 mm.

### 2.2. Analysis of Field Data

**Figure 4:**
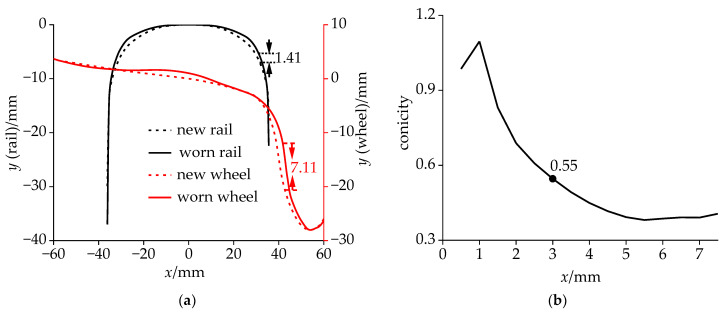
Wheel–rail relationship: (**a**) data of profile and (**b**) equivalent conicity.

The data of the rail and wheel profile in the test is shown in Figure 4, and the nominal rolling circle position was set as the zero on the *x*-axis. The curve of the worn rail shoulder was not as smooth as that of the new rail (CN60), and the inner side of the rail was 1.42 mm higher than the new rail at the coordinate of 31.1 mm. The wear of the wheel was mainly located in its flange and working area, with a maximum wear of 7.11 mm at the coordinate of 41.7 mm. The wear in the wheel working area was concentrated within the region of −10–20 mm on the *x*-axis, with a maximum wear of 1.1 mm. At the lateral displacement of 3 mm, the equivalent conicity reached 0.55, exceeding the reasonable range. According to the Klingel equation, the frequency of the hunting motion of a rigid constrained bogie can be expressed as follows:

(1) $$\omega_{t}=\frac{1}{2\pi }\sqrt{\frac{e}{dr_{0}}\left(\frac{d^{2}}{d^{2}+a_{b}^{2}}\right)}V$$

where *e* is the equivalent conicity; $a_{b}=1.25\;m$, and it represents half of the wheelbase; $r_{0}=0.46\;m$, and it represents the rolling circle radius; *d* = 0.7465 m, and it represents half of the rolling circle lateral distance on both sides of the wheelset; and *V* is the vehicle speed, expressed in km/h.

As can be inferred from Equation (1), the hunting frequency was directly proportional to both *e* and *V*. When *V* reached 350 km/h, $\omega_{t}=10.2\;\mathrm{Hz}$. In this scenario, the hunting frequency exceeded the DM frequency. It is noteworthy that, in the Klingel equation, the wheel profile is assumed to be conical. However, in reality, wheel profiles exhibit a worn-type shape. As the lateral displacement of the wheelset increases, the equivalent conicity further decreases, as illustrated in Figure 4b. Therefore, under actual operating conditions, the hunting frequency of the bogie is lower than $\omega_{t}$.

**Figure 5:**
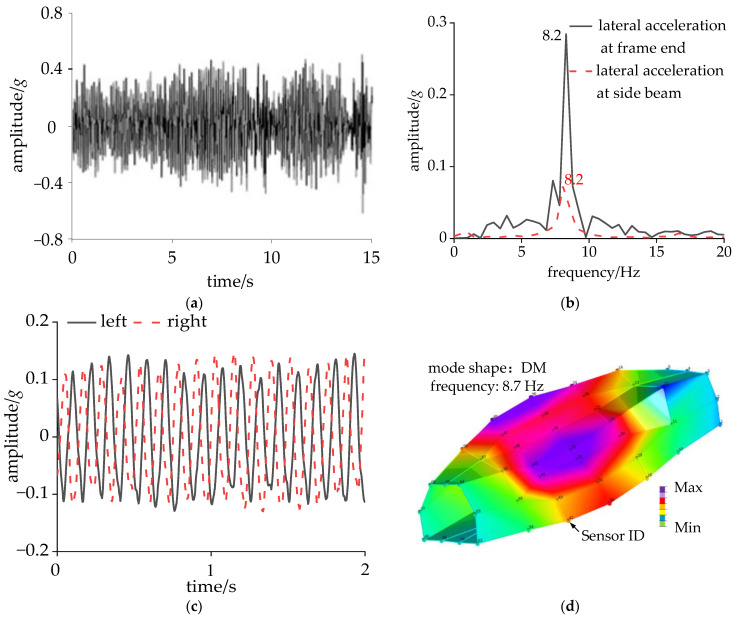
Data of accelerometers: (**a**) lateral acceleration at frame end in time domain; (**b**) lateral acceleration at frame end and side beam in frequency domain; (**c**) vertical acceleration at bolster in time domain; and (**d**) identification of DM.

Under CS conditions, the lateral acceleration of the frame end for 15 s is shown in Figure 5a. According to GB/T 5599 [32], a bandpass filter was applied to the acceleration at the frame end within the frequency range of 0.5–10 Hz. In the time domain, if the peaks of the acceleration value consecutively reach or exceed 8 m/s² six times, the bogie is classified as unstable. In Figure 5a, the maximum lateral acceleration at the frame end was 0.67*g*; hence, the bogie remained stable within this criterion. Figure 5b shows the results of the fast Fourier transform (FFT) of the lateral acceleration at the frame end and the side beam. There was a single main frequency vibration at both locations, with a frequency of 8.2 Hz, indicating that the bogie underwent a hunting motion of 8.2 Hz. Notably, the dominant frequency of the lateral vibration observed at the side beam coincided with that of the bogie, displaying an amplitude that was 25% of the bogie’s amplitude.

A band-pass filter with a frequency range of 0.5–20 Hz was applied to the vertical acceleration of the bolsters within 2 s. Figure 5c shows that the acceleration demonstrated obvious harmonic waves with opposite phases of vibration on the left and right sides of the bolster, suggesting that the cross-section of the carbody underwent a rolling motion in the vertical direction. The results of the operating modal analysis indicated that, under normal working conditions, the carbody DM frequency is 8.7 Hz, with the maximum deformation occurring at the middle junction of the side wall and roof. The lateral deformation of the floor displayed beam-like characteristics, with the most significant deformation situated at the side beam, which was symmetrical about the midsection in the longitudinal direction.

**Figure 6:**
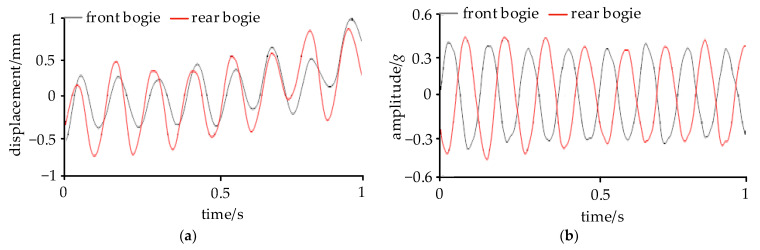
Phase analysis of bogie hunting: (**a**) displacement of YD and (**b**) lateral acceleration of frame end.

As illustrated in Figure 6, the maximum displacement of the YD sleeve approached 1 mm, with the longitudinal displacements of the front and rear YDs being nearly in-phase. Considering the installation direction of the YDs, it can be deduced that the phase difference of the relative yaw angles between the front and rear bogies and the carbody at the time of CS was close to 180°. The lateral accelerations at the frame end were opposite to each other, indicating that, at this moment, the front and rear bogies were in anti-phase hunting motion.

## 3. Simulation of Multi-Body Dynamics

### 3.1. Modeling of Rigid Flexible Coupled Dynamics

The carbody of the high-speed EMU in the preparation condition included an aluminum alloy carbody, interior decorations, and underframe equipment, with a total weight of 41.4 t. The materials were classified into four categories based on their location, and the parameters are shown in Table 2. The aluminum alloy carbody model was established through HYPERMESH (version 2021.1), and the meshing was performed using three-node shell elements (S3) and four-node shell elements (S4R), with a total mass of approximately 10.6 t. The interior decorations were attached to the carbody in the form of mass units, with a total mass of approximately 20.5 t. The four pieces of underframe equipment, excluding the tractive transformer (transformer), had a combined mass of 3.8 t and were rigidly connected to the floor. The transformer had a mass of 6.5 t and was elastically connected to the middle of the carbody. The transformer had four suspension points, which were displaced on the side beam.

The longitudinal stiffness between the transformer and the carbody was relatively high, with the main focus on stabilizing its longitudinal motion. The later and vertical suspension frequency of the transformer were as follows:

(2) $$\left\{ \begin{array}{l} f_{ey}=\frac{1}{2\pi }\sqrt{\frac{K_{ey}}{M_{e}}}\\ f_{ez}=\frac{1}{2\pi }\sqrt{\frac{K_{ez}}{M_{e}}} \end{array}\right.$$

where $M_{e}$ is the mass of the transformer, and $K_{ey}$ and $K_{ez}$ are the total stiffnesses of the lateral and vertical suspension, respectively.

**Figure 7:**
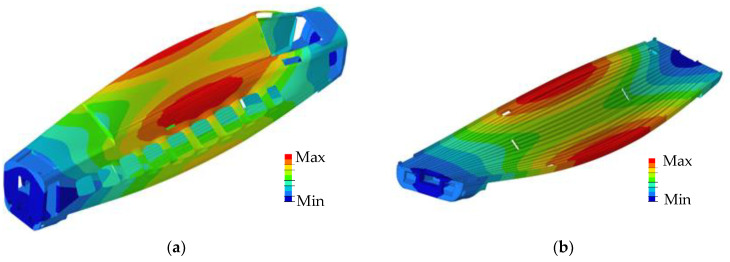
DM deformation: (**a**) overview and (**b**) floor.

Under the finite element (FE) model, the first 15 modes of the carbody were calculated, yielding a maximum modal frequency of 23.8 Hz. The low-order modes mainly included the first-order VBM, the first-order DM, the second-order VBM, etc. When performing a modal analysis with ABAQUS (version 2020), the vast number of the nodes and degrees of freedom contained in the calculation results can significantly prolong the simulation time. To simplify the model and enhance the computational efficiency, the dynamic reduction method [33] was employed, where measuring points on the floor and points for force applications on the bolster were selected as interface nodes. Within the FEMBS interface of SIMPACK (version 2021x), the substructure modal data were converted into an fbi file, which represents the flexible body file. Our research was centered on the first-order DM, specifically noting that its overall frequency was 8.7 Hz when the transformer was included in the carbody model, as shown in Figure 7.

The result of the modal analysis is presented in Table 3. The maximum frequency error between the FE model and the fbi file was 3.6%, which met the precision requirements of the simulation.

**Figure 8:**
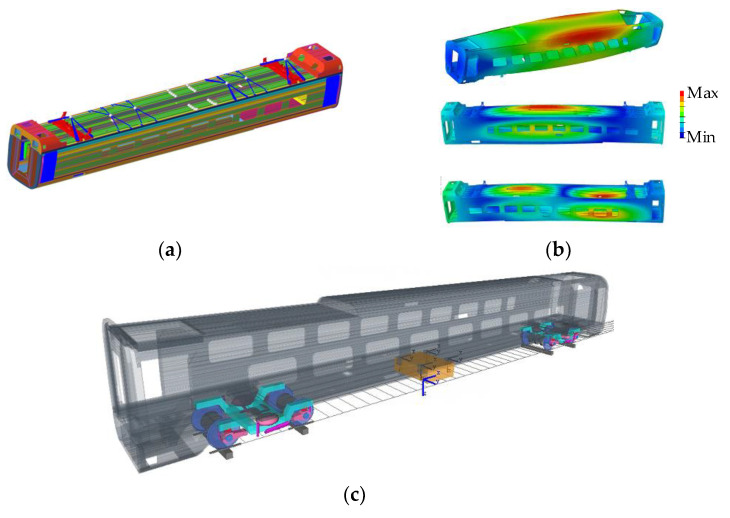
Procedure of vehicle dynamics modeling: (**a**) FE model; (**b**) modal analysis; and (**c**) SIMPACK model.

In the SIMPACK model, the profile of the wheel and the rail came from the field measurements in Figure 4a. The carbody was sourced from the flexible body file, while the other components were all considered rigid bodies. Figure 8c reveals that the model consisted of two frames, four wheelsets, and two traction rods, with each containing six degrees of freedom. Each bogie had four axle boxes with one degree of freedom around the axle.

### 3.2. Reproduction and Analysis of CS

**Figure 9:**
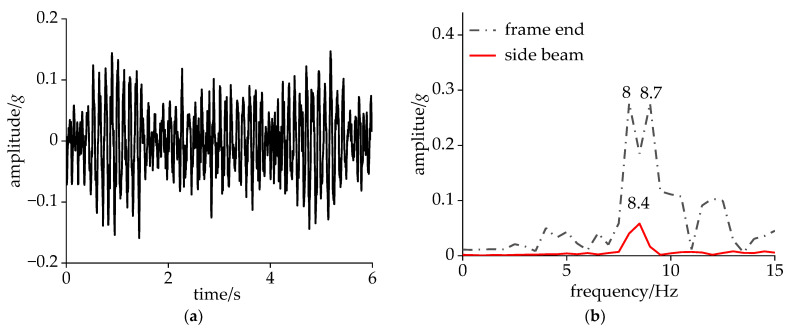
Lateral acceleration at design speed: (**a**) side beam in time domain and (**b**) frame end and side beam in frequency domain.

The simulation utilized the measured track irregularity data from the Beijing–Tianjin line, with the vehicle speed set as 350 km/h. As shown in Figure 9a, it was evident that the lateral acceleration of the side beam exhibited a vibration cycle within 0–1.5 s, with the vibration amplitude exceeding 0.11*g* during 0.5–1.4 s and peaking at 0.14*g* before attenuating. In contrast, the test results showed harmonic vibration of an equal amplitude in the carbody’s lateral acceleration, with amplitudes of around 0.1*g*. Figure 9b indicates that the dominant frequency of the carbody’s lateral acceleration was 8.4 Hz, which was generally close to the test results. Additionally, the lateral acceleration at the frame end exhibited peaks at 8 Hz and 8.7 Hz, with an amplitude of 0.28*g*.

**Figure 10:**
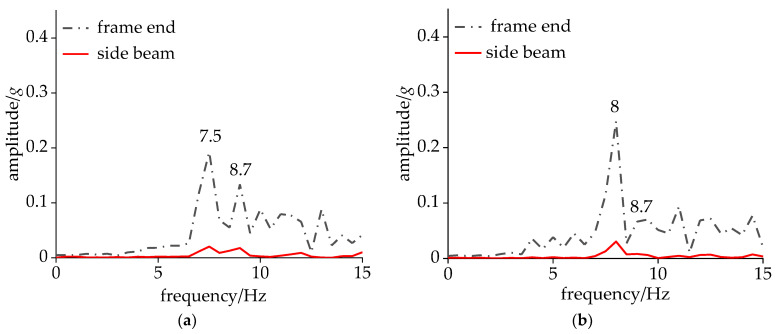
Acceleration under non-shaking conditions: (**a**) 280 km/h and (**b**) 290 km/h.

To further investigate the vibration frequency spectrum characteristics of the carbody and bogie during CS, a dynamic simulation was conducted with the vehicle’s operating speed incrementally increased from 280 km/h. As depicted in Figure 10, the acceleration amplitude of the side beam was relatively small, and both the carbody and the frame shared the same dominant frequencies within the range of 7–10 Hz. Among these two dominant frequencies, 8.7 Hz corresponded to the DM, while the other frequency represented the hunting frequency of the bogie. When the vehicle speed reached 290 km/h, the hunting frequency at the frame end increased by 0.5 Hz, accompanied by an amplitude increase to 0.25*g*, while the amplitude of the DM vibration decreased by 0.06*g*. At the speed of 295 km/h, multiple frequencies emerged within the 7–10 Hz range for the bogie, with 8.4 Hz being the frequency associated with CS. As the speed continued to increase, both the bogie and the carbody exhibited a single dominant frequency of 8.4 Hz, with the carbody amplitude reaching 0.08*g*.

**Figure 11:**
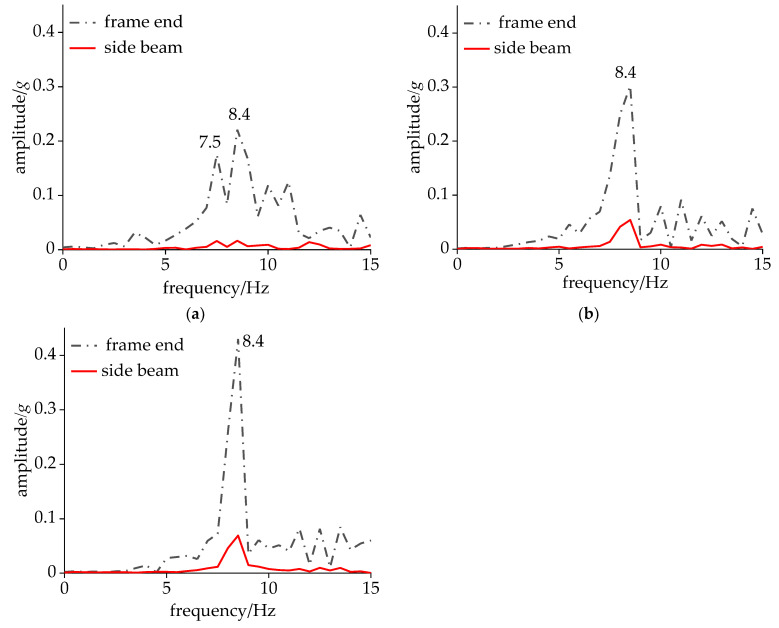
Acceleration under CS: (**a**) 295 km/h; (**b**) 300 km/h; and (**c**) 320 km/h.

Drawing insights from Figure 9b and Figure 11, it is evident that, within the speed range of 295–350 km/h, the frequency of the CS remained at 8.4 Hz, which was 0.3 Hz lower than the DM frequency of the carbody. Specifically, within the narrower range of 295–320 km/h, the frame end showed a single shaking frequency. However, as the velocity escalated further, the dominant frequency at the frame end bifurcated into two distinct peaks, embodying the frequency of bogie hunting and DM.

In the measured equivalent conicity curve, when the lateral displacement of the wheel exceeded 5 mm, the conicity decreased to below 0.39, as shown in Figure 4b. In this case, the hunting frequency will decrease, so the dynamic simulation results were consistent with the field test results. Therefore, when optimizing the suspension parameters of the equipment, the CS frequency considered was about 0.3 Hz lower than the DM frequency, and the new scheme should meet the requirement that the carbody has a smaller modal vibration amplitude at this frequency.

## 4. Analysis of CS Based on Modal Vibration Equation

### 4.1. Simplified Method for DM

**Figure 12:**
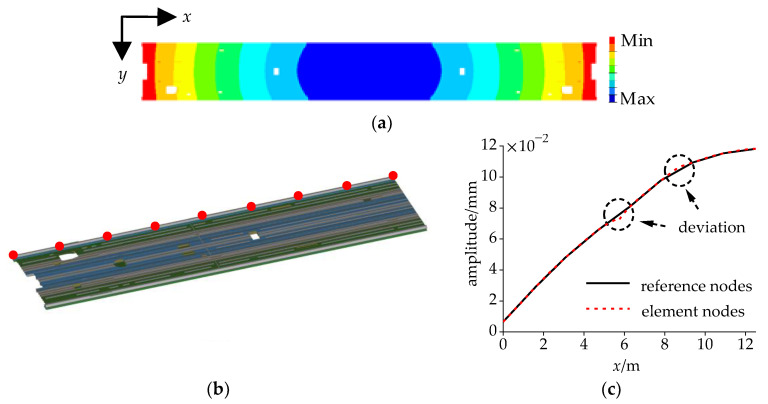
Lateral deformation amplitude of the floor: (**a**) cloud map; (**b**) selection of reference nodes; and (**c**) amplitude of the nodes.

The lateral deformation of the floor under the DM is shown in Figure 12a, where the deformation in the middle along the *x*-axis is greater than that at the ends, and the deformation of the side beams along the *y*-axis is greater than that in the middle. The deformation is approximately symmetrical about the center along both the *x*-axis and *y*-axis.

Half of the side beam had a total of 625 element nodes. As demonstrated in Table 4, the lateral deformation amplitude of these element nodes was extracted and nine reference nodes were selected at equal intervals. Figure 12c indicates that the amplitude at the ends was the smallest, but was still greater than zero. Linear connections were established between adjacent reference nodes. Within the range of 0–4 m, the deviation between the curves of the element nodes and the reference nodes was relatively small, and the slopes of the curves were steep. However, within the range of 4–12.5 m, the slopes of the curves became gentler, and significant deviations between the curves of the element nodes and the reference nodes occurred at 5.7 m and 8.5 m, with the maximum deviation reaching $3.1\times 10^{–3}$ mm, which translates to an error of 4.1%. Therefore, the accuracy of the lateral mode amplitude in the simplified model satisfied the requirements of the simulation.

The DM motion can be represented as the product of the modal coordinates (*T*) and the mode shape (*H*). *T* is a time-dependent variable and *H* is the inherent characteristic of the mode. The lateral mode shape function for the diamond mode can be written as follows:

(3) $$H_{n}\left(x\right)=H_{a}\left(k_{n}x+b_{n}\right)$$

where $x\in \left(x_{n},x_{n+1}\right)$, *n* = 1–8, and $x_{n}$ and $H_{n}/H_{a}$ are the coordinate and amplitude of the reference nodes, respectively.

Assuming that the mass of the carbody is uniformly distributed along the *x*-axis, the coefficient $H_{a}$ can be obtained through a normalized vibration mode function, which can be expressed as follows:

(4) $$2\sum_{n=1}^{8}\int_{x_{n}}^{x_{n+1}}\frac{M_{c}}{L}H_{n}^{2}dx=1$$

where $M_{c}$ and *L* are the mass and length of the carbody, respectively.

### 4.2. Vibration Contribution of Secondary Suspension Forces

**Figure 13:**
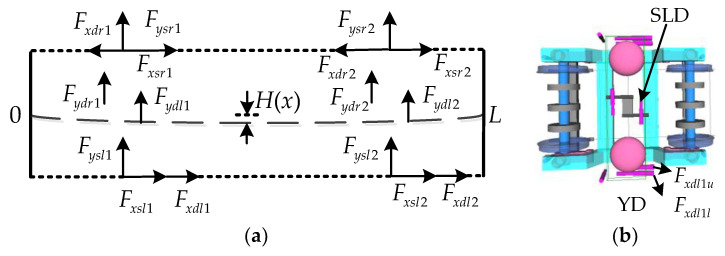
Force analysis of DM vibration: (**a**) forces and points of action and (**b**) installation of the damper.

Figure 13a shows the forces involved in the DM vibration of the carbody, where $F_{ysl1}$, $F_{ysr1}$, $F_{ysl2}$, and $F_{ysr2}$ are the lateral forces of the air spring; $F_{xsl1}$, $F_{xsr1}$, $F_{xsl2}$, and $F_{xsr2}$ are the longitudinal forces of the air spring; $F_{ydl1}$, $F_{ydr1}$, $F_{ydl2}$, and $F_{ydr2}$ are the lateral forces of the secondary lateral dampers (SLDs); and $F_{xdl1}$, $F_{xdr1}$, $F_{xdl2}$, and $F_{xdr2}$ are the longitudinal forces of the YDs. Although the DM vibration is a coupled vibration in the roll and lateral directions, the theoretical model here only considered the lateral direction of the carbody, and the anti-roll torsion bar was not involved. In addition, the anti-roll torsion bar mainly affected the rigid roll frequency of the carbody, and had little effect on vibrations above 8 Hz. Therefore, there was no anti-roll torsion bar in the theoretical model. The carbody did not adhere to the lateral stop in a straight line, so the force of the lateral stop was ignored. The modal vibration equation of the DM can be expressed as follows:

(5) $$\begin{array}{c} \ddot{T}+2\omega_{d}\zeta_{d}\dot{T}+\omega_{d}^{2}T=-\sum_{j=1}^{2}\left(F_{ysrj}+F_{yslj}\right)H\left(x_{j}\right)-\sum_{j=1}^{2}\left[F_{ydlj}H\left(x_{j}\right)+F_{ydrj}H\left(x_{j}\right)\right]\\ -\sum_{j=1}^{2}\left(F_{xsrj}-F_{xslj}+F_{xdrj}-F_{xdlj}\right)d_{3}\frac{dH\left(x_{j}\right)}{dx} \end{array}$$

where $\omega_{d}$ and $\zeta_{d}$ are the frequency and damping ratio of the DM, $x_{j}$ (*j* = 1–2) is the coordinate of the second suspension position, the amplitude of the DM at the front and rear SLD of the same bogie was determined to be the same ($H\left(x_{j}\right)$), and $d_{3}$ is half of the lateral distance of the secondary suspension.

The time-domain diagram of the output force of the left YD ($F_{xdl1u}$ and $F_{xdl1l}$), air spring ($F_{xsl1}$ and $F_{ysl1}$), and SLD ($F_{ydr1}$) of the front bogie is shown in Figure 13a. The maximum amplitude of $F_{ydr1}$ could reach 1500 N, and the output forces of $F_{xdl1u}$ and $F_{ysl1}$ were almost equal, with a maximum amplitude of 6000 N. In contrast, $F_{xsl1}$ and $F_{xsl1}$ were very small, and their contribution to the DM vibration was not significant.

**Figure 14:**
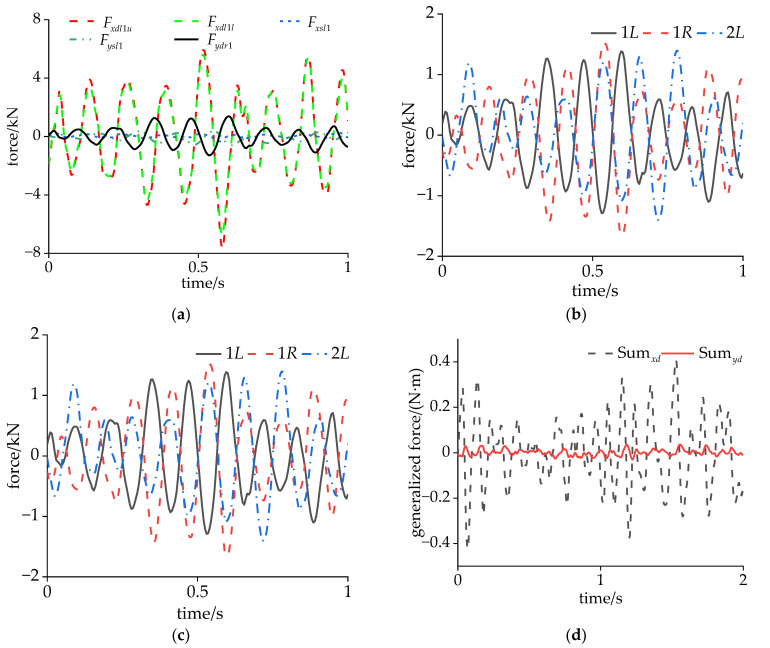
Comparison of output forces during CS: (**a**) forces at the front bogie; (**b**) forces of the SLD; (**c**) forces of the YD; and (**d**) total modal generalized forces.

From Figure 14b, it can be seen that the phase of $F_{ydr1}$ and $F_{ydr1}$ was close to 180°, and at this time, the two forces cancelled each other in the modal vibration equation. 

The forces of the YDs on the left and right sides of the same bogie acted in the form of a torque in the modal vibration equation. According to Equation (5), the sum of the modal generalized forces (MGFs) of the YDs and the SLDs are defined as ${\mathrm{Sum}}_{xd}$ and ${\mathrm{Sum}}_{yd}$. According to the symmetry of the DM, the MGF can be written as follows:

(6) $$\left\{ \begin{array}{l} {\mathrm{Sum}}_{xd}=\left(F_{xdr1}-F_{xdl1}-F_{xdr2}+F_{xdl2}\right)d_{3}\frac{dH\left(x_{1}\right)}{dx}\\ {\mathrm{Sum}}_{yd}=\left(F_{ydr1}+F_{ydl1}+F_{ydr2}+F_{ydl2}\right)H\left(x_{1}\right) \end{array}\right.$$

Based on the position of the force application and the amplitude of the DM, it can be seen that ${\mathrm{Sum}}_{xd}$ is much greater than ${\mathrm{Sum}}_{yd}$, which has the greatest impact on the DM vibration. The variation pattern of ${\mathrm{Sum}}_{xd}$ is also consistent with the acceleration of the carbody. When the phase difference between the hunting of the front and rear bogies is 180°, the generalized force is the largest. Around 0.3–0.8 s, the hunting of the front and rear bogies approached the same phase, resulting in a smaller MGF and a smaller acceleration of the carbody.

### 4.3. Optimization of Transformer Suspension Parameters

Referring to the mechanical model of DVA, the lateral coupled vibration model of the transformer and the carbody DM included two degrees of freedom, namely the lateral motion of the transformer and the DM motion.

**Figure 15:**
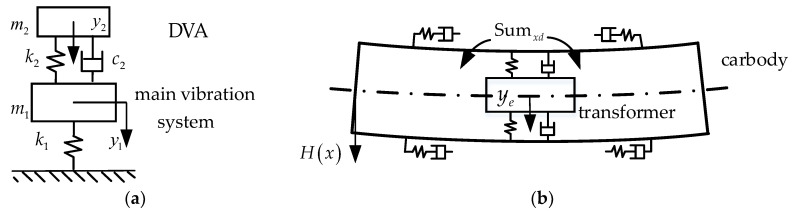
DVA design for the transformer: (**a**) mechanical model and (**b**) lateral coupled vibration model of the transformer and the DM.

In Figure 15a, the main vibration system, with a mass of $m_{1}$, exhibits a vertical degree of freedom represented by $y_{1}$, and is suspended from the foundation by a spring with a stiffness of $k_{1}$. The DVA has a mass of $m_{2}$ and is suspended with a stiffness of $k_{2}$ and a damping coefficient of $c_{2}$. The vibration differential equation of the system can be expressed as follows:

(7) $$\left\{ \begin{array}{l} m_{1}{\ddot{y}}_{1}+k_{1}y_{1}=k_{2}\left[y_{2}-y_{1}\right]+c_{2}\left[{\dot{y}}_{2}-{\dot{y}}_{1}\right]+F_{0}\\ m_{2}{\ddot{y}}_{2}=k_{2}\left[y_{1}-y_{2}\right]+c_{2}\left[{\dot{y}}_{1}-{\dot{y}}_{2}\right] \end{array}\right.$$

where the excitation force can be written as $F_{0}=e^{i\omega t}$.

By performing the Laplace transform on Equation (7), the system’s response can be expressed as follows:

(8) $$\left[\begin{array}{l} y_{1}\left(\omega \right)\\ y_{2}\left(\omega \right) \end{array}\right]=\frac{1}{-\omega^{2}M+i\omega C+K}\left\{\begin{array}{c} F\left(\omega \right)\\ 0 \end{array}\right\}$$

where M, C, and K are the mass, damping, and stiffness matrices of the system, respectively.

The displacement transmissibility ratio ($A_{g}\left(\omega \right)$) is defined as the vibration amplitude of $y_{1}$ to the static deflection ($1/k_{1}$), which can be expressed as follows:

(9) $$A_{g}\left(\omega \right)=\sqrt{\frac{{\left(1-C\omega^{2}\right)}^{2}+F^{2}{\left(\zeta \omega \right)}^{2}}{{\left(A\omega^{4}-B\omega^{2}+1\right)}^{2}+{\left(E-D\omega^{2}\right)}^{2}{\left(\zeta \omega \right)}^{2}}}$$

where $A=C=1/\gamma^{2}$, $B=1+u+1/\gamma^{2}$, $D=2(u+1)/\gamma^{2}$, $E=F=2/\gamma^{2}$, $u=m_{2}/m_{1}$, $\gamma =\sqrt{k_{2}/m_{2}}/\left(2\pi \omega_{1}\right)$, and $\zeta =c_{2}/\left(2m_{2}\omega_{1}\right)$.

**Figure 16:**
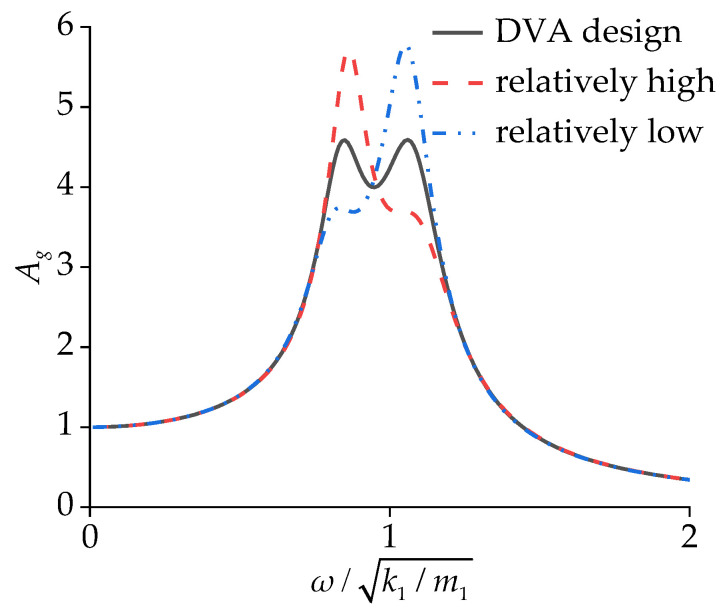
Influence of $k_{2}$ on displacement transmissibility ratio.

In the two-degrees-of-freedom system shown in Figure 15a, the curve of $A_{g}$ features two prominent peaks. As shown in Figure 16, an improper selection of $k_{2}$ can actually amplify the peaks in the curve. When $k_{2}$ is relatively high, the peak representing the equipment suspension frequency in the curve is pronounced. Conversely, with a lower $k_{2}$, the peak corresponding to the DM frequency stands out in the curve. In the case of CS, the transformer’s original suspension frequency was relatively low, making it difficult to effectively suppress the DM vibration. The DVA design aims to reduce the amplitude of $y_{1}$; in other words, the peak values of $A_{g}$ are minimized under broadband excitation.

The suspension parameters of the DVA can be obtained from the following formula [34]:

(10) $$\left\{ \begin{array}{l} \frac{\sqrt{k_{2}/m_{2}}}{\sqrt{k_{1}/m_{1}}}=\frac{1}{1+u}\\ \frac{c_{2}}{2m_{2}\sqrt{k_{1}/m_{1}}}=\sqrt{\frac{3u}{8{\left(1+u\right)}^{3}}} \end{array}\right.$$

The lateral coupled vibration equation for the DM and the transformer can be formulated as follows:

(11) $$\left\{ \begin{array}{l} \ddot{T}+{\omega_{d}}^{2}T=K_{ey}\left[y_{e}-H\left(L_{e}\right)T\right]H\left(L_{e}\right)+C_{ey}\left[{\dot{y}}_{e}-H\left(L_{e}\right)\dot{T}\right]H\left(L_{e}\right)+F_{0}\\ M_{e}{\ddot{y}}_{e}=K_{ey}\left[H\left(L_{e}\right)T-y_{e}\right]+C_{ey}\left[H\left(L_{e}\right)\dot{T}-{\dot{y}}_{e}\right] \end{array}\right.$$

where $L_{e}$ is the coordinate of the transformer, $C_{ey}$ is the total damping of the lateral suspension, and $F_{0}$ is the MGF of the external excitation.

Optimizing the lateral suspension of the transformer involves improving its coupled vibration relationship with the carbody. The secondary suspension force is only used as the excitation source transmitted to the carbody. According to the analysis in Section 4.2, the main factor affecting carbody shaking is the yaw damper, and the influence of the lateral damper is relatively small. Therefore, in the theoretical model, the excitation source is the torque applied through the yaw damper, without considering the lateral damper. Therefore, $F_{0}$ can be expressed as follows:

(12) $$F_{0}={\mathrm{Sum}}_{xd}$$

To facilitate the solution for the optimal $K_{ey}$ and $C_{ey}$, $y_{1}$ in Equation (7) was replaced by $H\left(L_{e}\right)T$, and Equation (11) can be written as follows:

(13) $$\left\{ \begin{array}{l} \frac{\left[H\left(L_{e}\right)\ddot{T}\right]}{H^{2}\left(L_{e}\right)}+\frac{{\omega_{d}}^{2}\left[H\left(L_{e}\right)T\right]}{H^{2}\left(L_{e}\right)}=K_{e}\left[y_{e}-H\left(L_{e}\right)T\right]+C_{e}\left[{\dot{y}}_{e}-H\left(L_{e}\right)\dot{T}\right]+\frac{{\mathrm{Sum}}_{xd}}{H\left(L_{e}\right)}\\ M_{e}{\ddot{y}}_{e}=K_{e}\left[H\left(L_{e}\right)T-y_{e}\right]+C_{e}\left[H\left(L_{e}\right)\dot{T}-{\dot{y}}_{e}\right] \end{array}\right.$$

Therefore, the optimized frequency of the transformer was obtained as follows:

(14) $$f_{ey}=\frac{\omega_{d}}{1+M_{e}H{\left(L_{e}\right)}^{2}}$$

According to the theory of DVA, it is understood that the transformer can effectively absorb the DM vibration when its lateral suspension frequency is slightly lower than the DM frequency. In Equation (7), the effectiveness of the DVA is dependent on *u*. This implies that the CS suppression effect becomes more pronounced as both the mass of the transformer and the modal amplitude at the suspension position increase.

**Figure 17:**
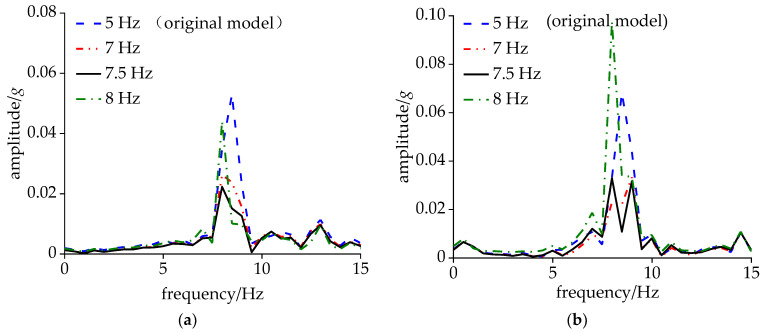
The influence of the lateral transformer suspension frequency on the acceleration of the side beam: (**a**) lateral and (**b**) vertical.

Figure 17 investigates the impact of $f_{ey}$ on CS, with the simulation conditions consistent with those in Section 3.2. The initial value of $f_{ey}$ was set at 5 Hz, with each subsequent increment being 0.5 Hz. The amplitude of the acceleration at the side beam was calculated. As $f_{ey}$ increased, the vertical and lateral acceleration at the side beam decreased simultaneously. When $f_{ey}$ was 7.5 Hz, the lateral acceleration amplitude of the side beam was 0.022*g* and the vertical amplitude was 0.033*g*, which are, respectively, 41.5% and 48.5% of the values under the original parameters. At this point, the acceleration amplitude at the side beam was the smallest. When $f_{ey}$ was above 7.5 Hz, the acceleration amplitude at the side beam increased rapidly. Therefore, the optimized lateral suspension frequency of the transformer was set to 7.5 Hz.

Strictly speaking, the side beam is not the location for measuring the ride comfort index in GB/T 5599, but the vibration amplitude at this position on the floor is the largest. Therefore, the ride comfort index at the floor center and side beam before and after optimization was calculated.

**Figure 18:**
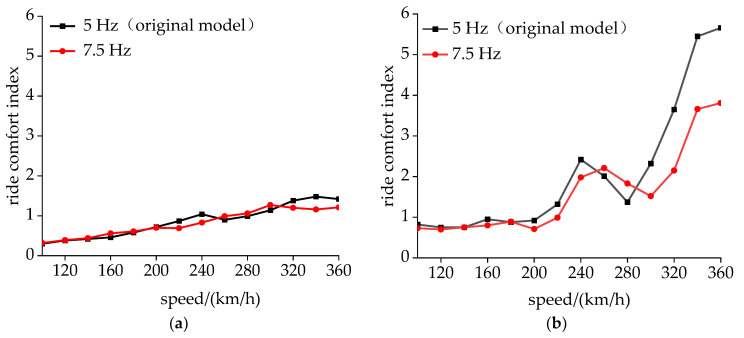
Comparison of ride comfort index at different speeds: (**a**) floor center and (**b**) side beam.

**Figure 19:**
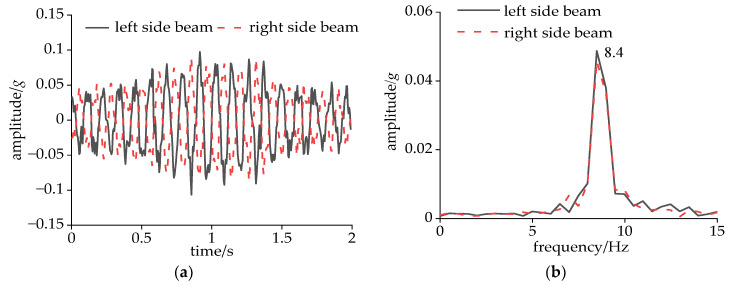
Vertical acceleration of the carbody at 240 km/h in the original model: (**a**) time domain and (**b**) frequency domain.

As can be seen from Figure 18a, the ride comfort index at the floor center remained at a low value due to the smaller amplitude of the DM there. After optimization, the ride comfort index at the floor center decreased slightly. This decrease was relatively significant at speeds of 240 km/h and 350 km/h. In Figure 18b, it can also be observed that the comfort index had a peak at 240 km/h and quickly decreased within the range of 240–280 km/h. A detailed analysis of the acceleration experienced by the bolster beams at a speed of 240 km/h is presented in Figure 19. Notably, both side beams exhibited pronounced harmonic vibrations with a phase difference approaching 180, indicating an opposing vibrational pattern. Figure 19b reveals that the dominant frequency of the side beam accelerations aligned precisely with the CS frequency, with an amplitude magnitude of 0.049*g*. Unlike CS, this abnormal vibration rapidly decayed with increasing speed, indicating that a specific wavelength in the track irregularity at the current speed excited the DM of the carbody.

As shown in Figure 18b, when the vehicle speed reached 280 km/h in the original model, the ride comfort index at the side beam increased significantly, corresponding to the CS. After optimization, the vehicle speed that triggers CS was increased by 10 km/h, and the ride comfort index at 350 km/h was 67% of the original model.

## 5. Conclusions

Starting with the field test, this paper analyzed the acceleration characteristics of a carbody and bogie under CS. Based on the modal vibration equation, the contribution of suspension forces to CS was analyzed. The lateral suspension frequency of the transformer was optimized according to the amplitude of the side beam acceleration. The conclusions obtained are as follows:

(1) When carbody shaking occurs, the wheels are at the end of their maintenance cycle and the rails are in an under-polished state. The abnormal wheel–rail relationship can cause the high-frequency hunting of the bogie, which can excite the diamond mode. Relevant operational departments should promptly carry out maintenance on the wheels and rail.
(2) During carbody shaking, the main contribution to the modal vibration of the vehicle body among the secondary suspension forces originated from the yaw dampers, with the hunting motion of the front and rear bogies being in opposite phases. Further research can be conducted based on these conclusions to develop active control strategies for suppressing carbody shaking.
(3) When the lateral suspension frequency of the transformer was adjusted to 7.5 Hz, the reduction in the acceleration at the midsection of the side beam was the greatest, and the comfort index at this location was reduced by 33% at the speed of 350 km/h. Since the shaking of the carbody in the lateral and vertical directions are coupled, optimizing the suspension parameters in multiple directions can further enhance the vibration absorption capability of the underframe equipment.

## Figures and Tables

**Table 1:** Parameters of the sensors.

Type	Installation Position	Measuring Range	Sample Rate	Voltage Sensitivity
Accelerometers	Carbody	0–2*g*	1000 Hz	310 mV/*g*
	Bogie frame	0–18*g*	2000 Hz	100 mV/*g*
Laser transducer	Yaw damper	30–130 mm	2000 Hz	40 mV/*g*

**Table 2:** Material information of the carbody.

Material ID	Location	Density/(t/m^3^)	Elastic Modulus/GPa	Poisson’s Ratio
1	Roof	7.42	80	0.33
2	Floor	8.9	66	0.33
3	Side wall	8.9	135	0.33
4	End wall	5.4	69	0.33

**Table 3:** Results of modal analysis.

Order	Name	FE Frequency/Hz	fbi Frequency/Hz	Error
1	1st-order DM	9.10	9.66	0.66%
2	1st-order VBM	10.37	10.52	1.45%
3	2nd-order VBM	12.51	12.88	2.96%
4	2nd-order DM	12.61	13.07	3.65%
5	Local mode of the roof and floor	13.89	13.98	0.65%

**Table 4:** Deformation of reference nodes in FE calculation.

***x*/m**	0	1.6	3.1	4.7	6.3	7.8	9.4	10.9	12.5
**amplitude/mm**	0.0065	0.0290	0.0482	0.0658	0.0812	0.0977	0.1094	0.1153	0.1182

## Data Availability

The data are contained within the article.
